# Capturing the fast-food landscape in England using large-scale network analysis

**DOI:** 10.1140/epjds/s13688-018-0169-1

**Published:** 2018-10-17

**Authors:** Magda Baniukiewicz, Zachariah L. Dick, Philippe J. Giabbanelli

**Affiliations:** 1 Department of Computer Science, Northern Illinois University, DeKalb, USA; 2 Computer Science Department, Furman University, Greenville, USA

**Keywords:** Network science, Obesity, Spatial networks, Zoning

## Abstract

Fast-food outlets play a significant role in the nutrition of British children who get more food from such shops than the school canteen. To reduce young people’s access to fast-food meals during the school day, many British cities are implementing zoning policies. For instance, cities can create buffers around schools, and some have used 200 meters buffers while others used 400 meters. But how close is too close? Using the road network is needed to precisely computing the distance between fast-food outlets (for policies limiting the concentration), or fast-food outlets and the closest school (for policies using buffers). This estimates how much of the fast-food landscape could be affected by a policy, and complementary analyses of food utilization can later translate the estimate into changes on childhood nutrition and obesity. Network analyses of retail and urban forms are typically limited to the scale of a city. However, to design national zoning policies, we need to perform this analysis at a national scale. Our study is the first to perform a nation-wide analysis, by linking large datasets (e.g., all roads, fast-food outlets and schools) and performing the analysis over a high performance computing cluster. We found a strong spatial clustering of fast-food outlets (with 80% of outlets being within 120 of another outlet), but much less clustering for schools. Results depend on whether we use the road network on the Euclidean distance (i.e. ‘as the crow flies’): for instance, half of the fast-food outlets are found within 240 m of a school using an Euclidean distance, but only one-third at the same distance with the road network. Our findings are consistent across levels of deprivation, which is important to set equitable national policies. In line with previous studies (at the city scale rather than national scale), we also examined the relation between centrality and outlets, as a potential target for policies, but we found no correlation when using closeness or betweenness centrality with either the Spearman or Pearson correlation methods.

## Introduction

Road networks are one of the oldest forms of human-made infrastructure networks, preceding power and telecommunication networks. Before network science became a popular approach, geographers devoted several books to the analysis of road networks, including *Network Analysis in Geography* from the late 1960s [1] and the influential *The Seminal Logic of Space* in 1984 [2]. While some modern day cities may appear to have a grid-like pattern of roads, many road networks do not result from a central planning process but instead emerge over time as the result of an organic densification/exploration process [3] thus creating structures far more complex than square grids. Despite being shaped by local geographical and socio-economical factors, road networks also exhibit structural commonalities across cities and countries. For example, Buhl and colleagues found similar average degrees [4] while Cardillo *et al.* reported a fractal dimension (per the box-counting method) in the narrow 1.7–2.00 range [5]. For a summary of these commonalities, and a contextualization of findings among other spatial networks, we refer the reader to the review by Barthelemy [6].

Network science has been particularly interested in relating a network’s *structure* to its *function*. While there is a myriad of metrics, road networks are often analyzed with respect to betweenness centrality (since they are infrastructure networks and betweenness approximates traffic between all pairs of nodes) and closeness centrality (as a proxy to access). These metrics have been related to various phenomena, such as the presence of specific retail activities. In this paper, we focus on using the structure of road networks to understand the presence of fast-food outlets and its relation with the presence of schools. While analyses have been conducted on the structure of road networks at a large-scale [17, 18], studying their relation with retail activities has predominantly been at the city level (Table 1), which provides policymakers with information for a few selected cities but may not be sufficient for a national approach. Geographers and economists have also analyzed retail activities at a national level, but without using network-based metrics (e.g. shortest-paths calculations or centrality). For example, the geographical distribution of retail outlets was investigated at the scale of Sweden using buffers [19], while food deserts were examined in rural U.S. counties using county-level data [20], and economic activities across countries were assessed by imposing a square grid [21]. In this paper, we present the first large-scale analysis of retail activities (focusing on fast-food outlets) using network methods across the whole of England.

**Table 1:** Network science studies investigating various structures in road networks (sorted by year)

Ref.	Cities	Network Metrics	Phenomena
[7]	Bologna (Italy)	Centrality (closeness, betweenness, straightness)	Retail and service activities
[8]	Cambridge and Somerville, MA (USA)	Number of destinations available in a given radius (i.e. reach) and cumulative number of meters/turns/intersections to reach them using shortest paths; centrality (betweenness)	Retail activities, urban form, and land use
[9]	East Baton Rouge (USA)	Centrality (closeness, betweenness, straightness)	Land use
[10]	Barcelona (Spain)	Centrality (closeness, betweenness, straightness)	Retail activity
[11]	Edinburgh (Scotland), Leicester (England), Sheffield (England), Oxford (England), Worcester (England), Lancaster (England), Catania (Italy), Barcelona (Spain), Bologna (Italy), Geneva (Switzerland)	Centrality (closeness, betweenness, straightness, accessibility), street lengths, intersection angles, areas	Geometric properties
[12]	Neighborhoods of London (England)	Centrality (betweenness)	Gentrification
[13]	Stockholm (Sweden)	Centrality (closeness, betweenness, straightness)	Land use (built-up areas vs green areas)
[14]	Zhengzhou (China)	Centrality (closeness, betweenness, straightness)	Land use (Points Of Interests)
[15]	Cardiff (Wales)	Centrality (closeness, betweenness)	Property prices
[16]	Old cities (Kfar Saba, Raanana, Bat-Yam), new cities (Beer Sheva, Ashdod, Modiin), and hybrid cities (Lod, Ramle) in Israel	Degree, centrality (closeness, betweenness)	Retail activity

Studying the geography of fast-food outlets at a detailed level (e.g. using network metrics such as shortest-paths distances between outlets) over all of England is primarily motivated by the current public health context. In the United Kingdom (UK), based on the National Child Measurement Programme 2013–2014, one third of children aged 10–11 and over a fifth of those aged 4–5 were overweight or obese [22]. The current policy landscape in the UK emphasizes the role of eating patterns in achieving a healthy weight, and fast-food outlets have received particular attention. These outlets play a significant role in children’s nutrition: British secondary school children get more food from ‘fringe’ shops than from the school canteen [23]. In addition, even when there is a stay-on-site policy for lunch, the most popular time to buy food is after school [24]. This situation has led policymakers to increasingly advocate for the regulation of fast-food outlets as part of an overall strategy of obesity prevention in school neighbourhoods. Between 2011 and 2014, four reports have called for a restriction of fast-food outlets around schools [25–28]. However, policies have so far differed widely in design as they can restrict fast-food outlets (i) in terms of clustering clustering (e.g. minimum distance between them) or (ii) respectively to schools (e.g. with a minimum distance from schools) [29]. In addition, the impact of fast-foods on obesity and food consumption varies over space, and particularly depending on the deprivation of the area [30]. In this context, the principal contribution of the present work is to take a big data approach to propose the first investigation of fast-food activities based on road networks over an entire nation rather than on few select cities. Specifically, we conduct large-scale network analyses to:

1. contribute to the evidence base for coordinated regulation at the level of England by analyzing distances (i) between fast-food outlets and (ii) between fast-food outlets and schools, across deprivation levels.
2. investigate the relationship between centrality and the presence of fast-food outlets nation-wide, thus extending the scope of many previous studies employing network centrality mostly at the city-scale.

The remainder of this paper is divided into four sections. In Sect. 2, we summarize the different geographical layers in England and the associated datasets used for this study. In particular, we contextualize these datasets with respect to previous studies of food outlets in England, and we explain the different steps to pre-process the datasets. Pre-processing includes assigning fast-food outlets and schools to roads, building the road network, and identifying the deprivation level of each road segment. Our analysis methods (including centrality metrics and their computation) are summarized in Sect. 3, with results provided in Sect. 4. Results are discussed in Sect. 5 in terms of their contribution to the evidence-base for public health in England, and regarding the potential of using large-scale analyses to inform regulations going forward.

## Assembling a dataset

### Overview

**Figure 1:**
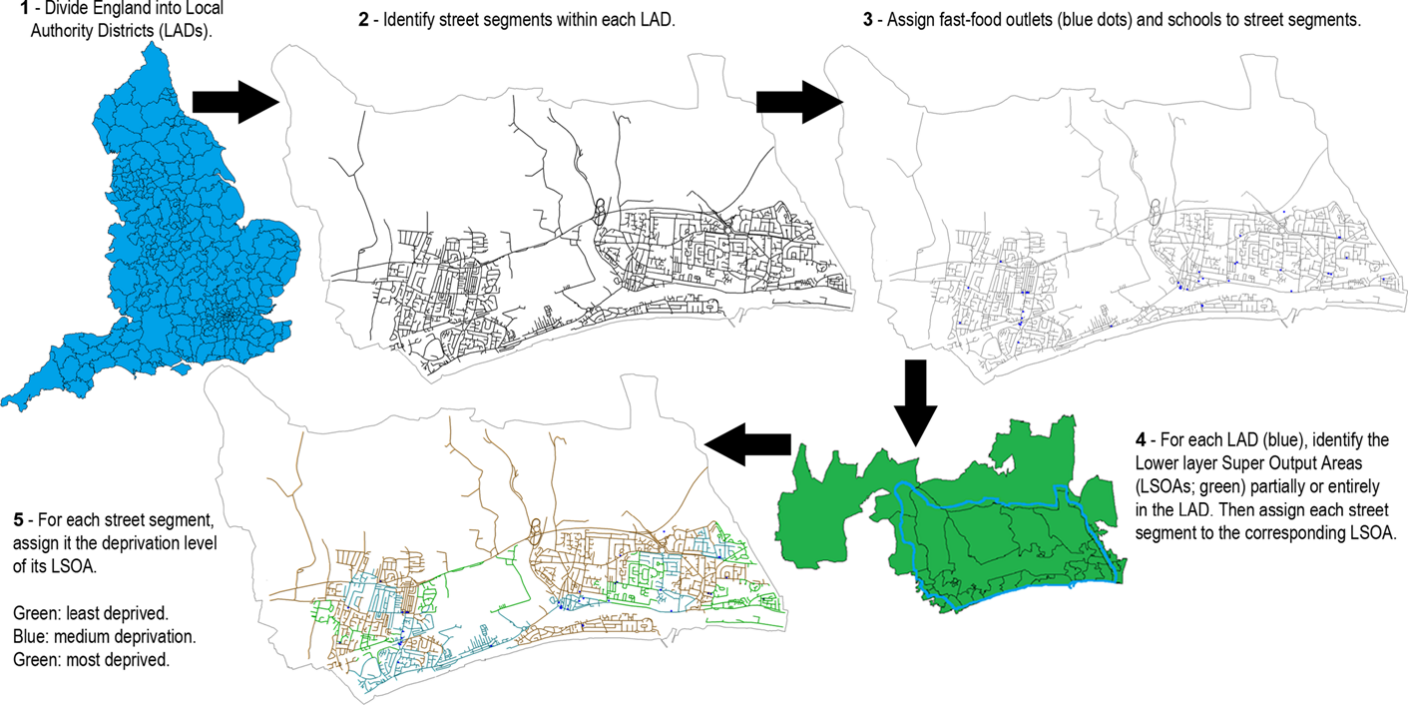
Our five steps process to combine five datasets into one, specifying the location of fast-food outlets and schools within road as well as the level of deprivation. *The high resolution version allows zooming to see detailed locations and deprivation levels within this sample LAD (Adur)*

Our objective was to assemble a data that includes the location of fast-food outlets and schools on the road network, and also provides the level of deprivation. This objective was accomplished in five steps, each involving the use of another dataset. We used a top-down process (Fig. 1), starting with the whole of England (thus excluding Wales, Scotland, and Northern Ireland) and dividing it into coarse units. Steps 1 divides England in Local Authority Districts (LADs), specified in the 2016 boundary line dataset. In step 2, we added in the 2016 Ordnance Survey (OS) Open Roads containing 3,396,694 roads. Specifically, we found the roads that resided (either entirely or partially) within each LAD. In step 3, we retrieved the location of fast-food outlets and schools from the Points of Interest (POI) data (PointX Database Right/Copyright 2016) obtained in January 2016. This dataset aggregates over 150 databases (in the ‘eating and drinking’ category) and has an accuracy ranging from 81% to 100% [30]. Locations for fast-food outlets were added to the street networks. At that stage, we had divided England into 327 LADs, each containing a road network, with fast-food outlets and schools assigned to each road segment. Although studies differ on how they measure deprivation, or the specific relation being deprivation and childhood obesity, they have often found a correlation between either deprivation and childhood obesity, or deprivation and the density of fast-food outlets (which also correlated with childhood obesity) [31–33]. Therefore, we also tracked deprivation using the official measure for small areas in England, the Index of Multiple Deprivation (IMD). This index takes into account employment, living environment, crime, health, education, income, and housing [34]. Tracking this score took two additional steps, because it was provided in datasets using different geographical units.

**Figure 2:**
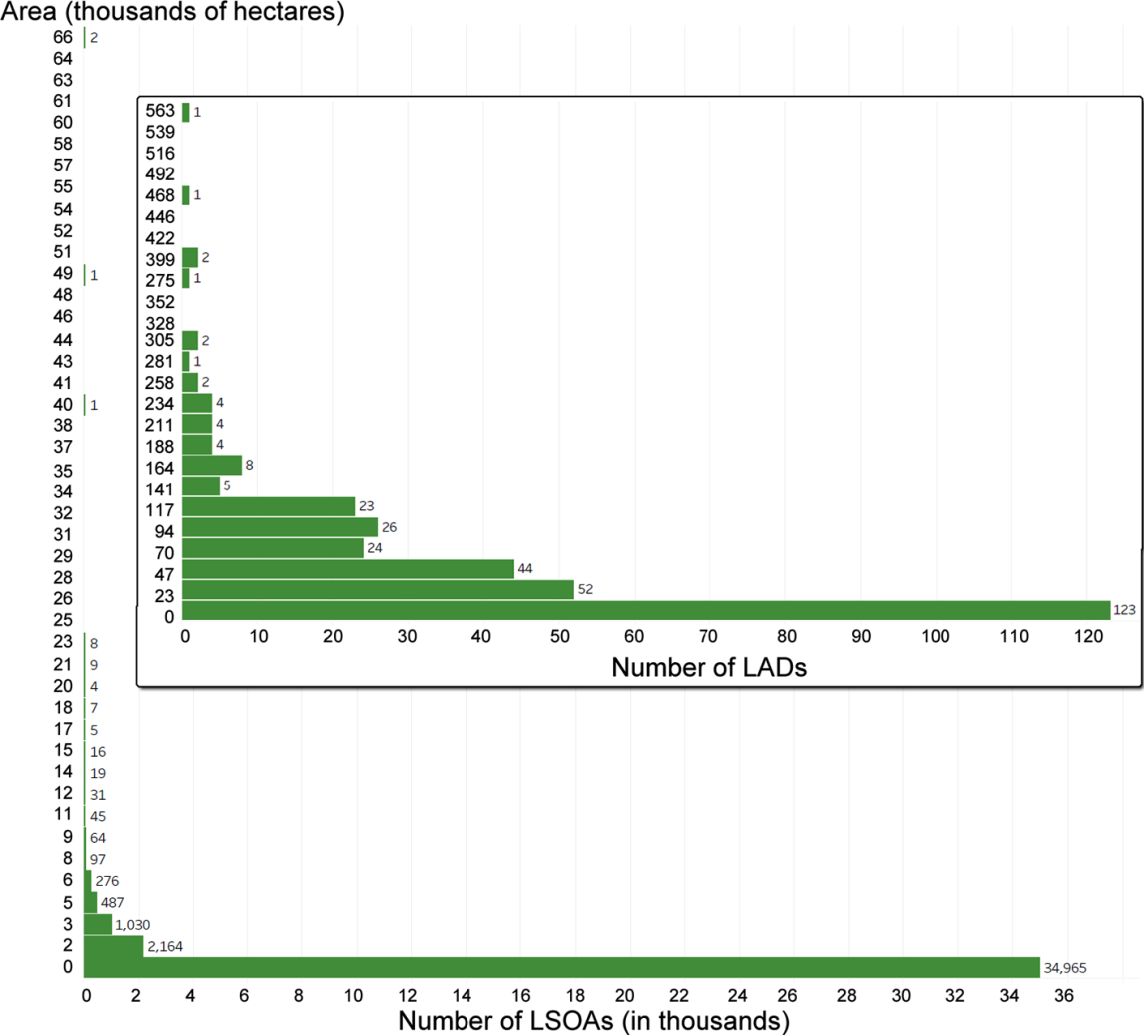
Distribution of sizes for LSOAs and LADs (inset), in thousands of hectares. The average LAD had 67,323 ± 78,264 hectares, while the average LSOA had 561 ± 1621 hectares

Whereas LADs are designed based on local governance, most statistics are available in census data, which uses different spatial units. England can be divided using three levels of spatial units, from largest to smallest: Middle layer Super Output Areas (MSOAs), Lower layer Super Output Areas (LSOAs), and Output Areas (OAs). The minimum and maximum number of inhabitants in each of these 3 possible subdivision is summarized in Table 2. In order to most accurately track deprivation levels, we used the most detailed level at which this information is available: LSOAs (Fig. 2). It should be noted that LSOAs provide a spatial resolution often used in studies of food geography focusing on a *single* city, such as Bristol [30], parts of Berkshire [35] or the North East of England [36]. However, using them in a *national* study (together with the whole road network) and conducting a detailed *network* analysis are two of the hallmarks of the present study, in contrast with previous work (Table 3).

**Table 2:** Minimum and maximum values for each subdvision type in the UK

Subdivision	Min value	Max value
OA	100 residents or 40 households	625 residents or 250 households
LSOA	1000 residents or 400 households	3000 residents or 1200 households
MSOA	5000 residents or 2000 households	15,000 residents or 6000 households

**Table 3:** Key features of previous studies of fast-food outlets in the UK

*Ref.*	*Area*	*Data*	*Analysis*
[37]	Norfolk county	The location of food-related outlets was extracted from the Yellow Pages directory issued in six years (1990, 1992, 1996, 2000, 2003, 2008). Locations were overlaid onto the 2001 electoral ward boundaries for Norfolk (*n* = 205)	Repeated measures analysis of variance (RMANOVA)/multiple logistic regression model
[38]	England and Scotland	The location of McDonald’s restaurants (*n* = 942) were obtained from the Yellow Pages directory and overlaid on 38,987 small areas: 6505 ‘Data zones’ in Scotland and 32,482 Super Output Areas in England	One-way analysis of variance
[30]	Avon county	The location of outlets was extracted from the Ordnance Survey Points of Interest in Avon county	Geographically weighted regression
[35]	Berkshire county	The location of outlets was obtained from six local councils, with analyses at the LSOA level	Cross-classified multi-level model with Markov chain Monte Carlo methods
[36]	North East of England	The location of food-related outlets was extracted from the Yellow Pages directory, with analyses at the LSOA level	Correlation analysis, logistic multinomial regression, ANOVA

In step 4, we used the latest (2011) census division of England into 34,753 LSOAs (which also included Wales). We removed Wales, and identified the LSOAs to which each road segment belonged. Finally, step 5 cross-referenced the LSOAs with the 2015 Indices of Multiple Deprivation dataset: since we knew the LSOA for each road, and the deprivation for each LSOA, we were able to assign a deprivation level for each road. The summary of datasets involved is provided in Table 4.

**Table 4:** Datasets combined for our study

*Step*	*Dataset*	*Year*	*Characteristics*
1	Boundary-Line^1^ products.html	2016	Shape files of polling districts, county and district regions, wards, etc. 1.41 Gb in total
2	Ordnance Survey (OS) Open Roads^2^	2016	3,396,694 roads
3	Points of Interest^3^	Jan. 2016	Location of 39,374 fast-food outlets and 25,755 schools
4	Lower Layer Super Output Area boundaries^4^	2011	Shape file of 34,753 geometries defining LSOAs
5	Indices of Multiple Deprivation^5^	2015	32,845 rows of IMD score and contributing elements (e.g., income, health)

This five step process required extensive data pre-processing, not only because of the sheer volume of information, but because of numerous challenges in combining the datasets (e.g., missing values, mismatch in geographical units). The operations involved in each step are now detailed, each within a dedicated sub-section. All of the scripts necessary to combine and pre-process the data are available within the ‘Pre-processing’ folder at https://osf.io/gn3f2/. Note that many of our spatial queries (e.g., to assess whether a road ‘fits’ within a LAD) require the open source library GeoTools for Java. As we do not own the data, links within Table 4 track data provenance.

### Step 1: dividing England into local authority districts (LADs)

Our process starts by using the 326 shape files defining LADs, from the boundary-line dataset. Note that each result is not only a geometry defining the boundaries of the LAD, but a spatial object due to the use of coordinates. It also has a name, which later steps use to double-check linking across datasets.

There are three important reasons to justify dividing England into LADs specifically. First, from a methodological standpoint, it allows to relate the structure of each city to the presence of fast-food outlets or schools. This point is detailed in Sect. 3.3 regarding centralities. Second, from a policy standpoint, while our study provides evidence across England, interventions are still conducted through local councils. Results thus need to be available at the city-level. Third, from an implementation standpoint, the division allows data parallelism: cities can be assigned to several computing cores for parallel computations.

### Step 2: finding the road segments within each LAD

**Figure 3:**
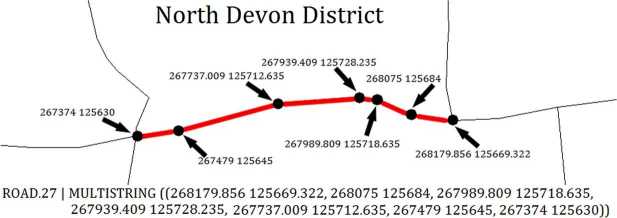
Roads are encoded in a shapefile as a series of segments. A segment links two points, specified as coordinates in easting and northing coordinates. Segments are created when a road has an intersection or turns

**Figure 4:**
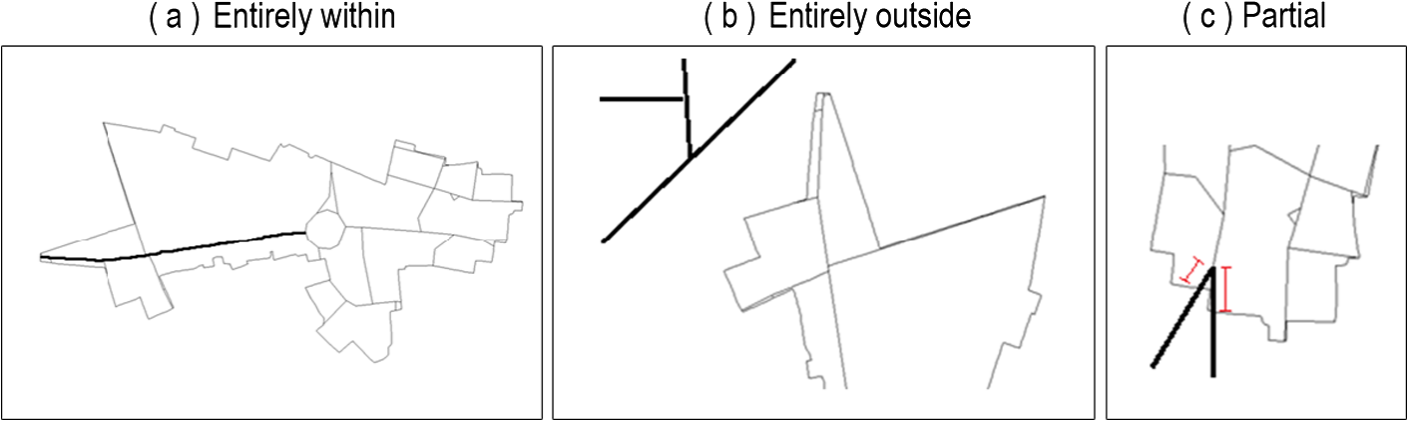
Three cases regarding the relationship between a road and an area

The input to step 2 consists of the output from step 1 (326 shape files for LADs) and the one shape file that defines roads as a series of segments, where new segments are made everytime a road bends or intersects with another road (Fig. 3). The output is a road *network*, divided across the LADs. To create this output, we need to (i) identify the (parts of) roads that belong to each LAD, and (ii) convert roads from a shapefile format into a network. For the identification, we go through each LAD, and then through each road. The trivial cases are when the road falls entirely outside the LAD (discarded), or entirely within (assigned to the LAD). The one intermediate case is when a *part* of a road falls within a LAD (Fig. 4). In this case, we divide the road in two segments: one segment for the LAD it belongs to (assigned to the LAD), and one remaining segment. Note that, while LADs do not overlap, some road segments may be at the border of two LADs. In this case, the segments are assigned to both LADs (i.e. duplicated). For the conversion, each road segment corresponds to one edge of our network, and each node stores the coordinates of the segment’s endpoints as in Fig. 3. Note that our edges are not a one-to-one mapping of road segments in the road shape file, because some road segments may be sub-divided when they span two LADs.

**Figure 5:**
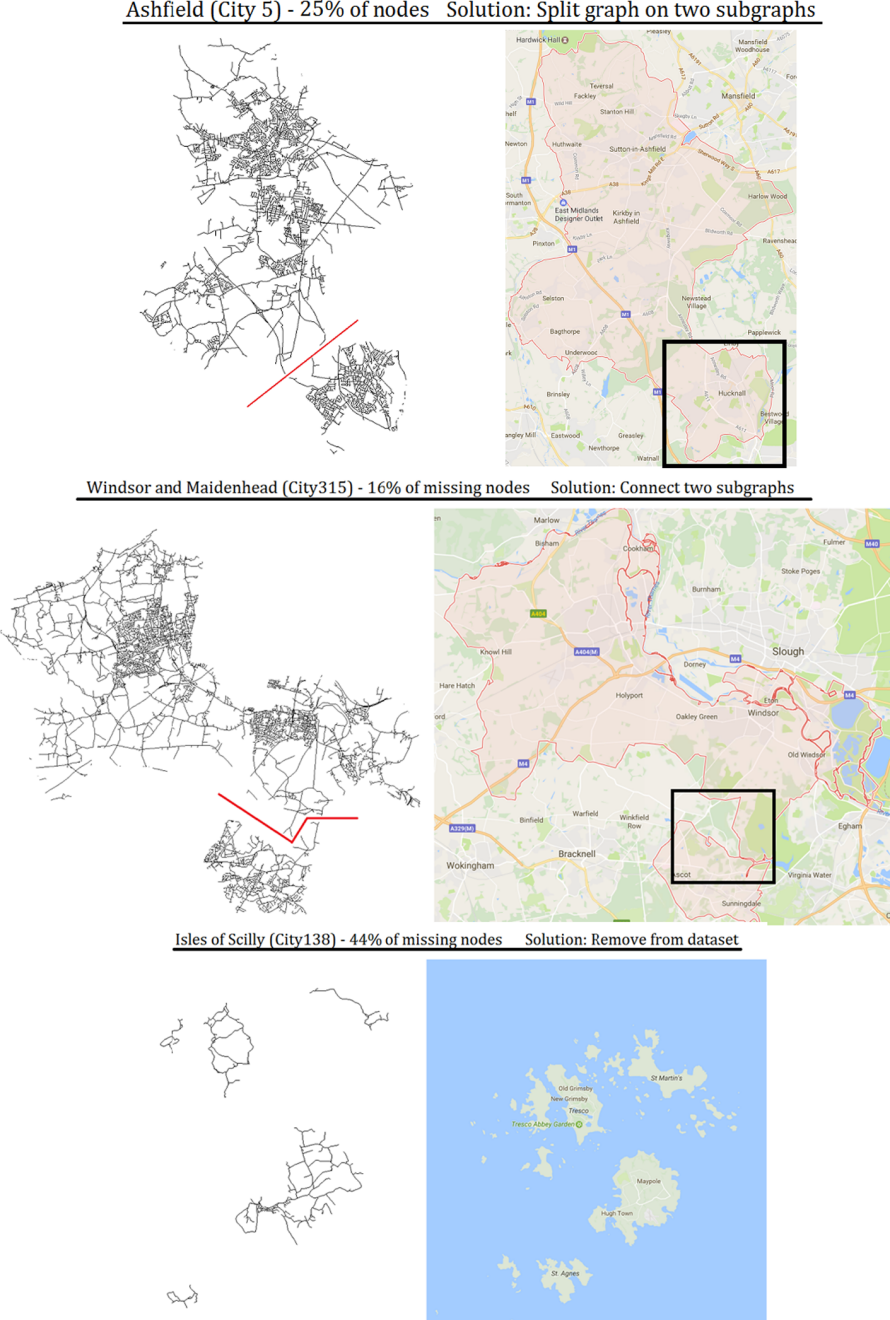
Three situations leading to a largely disconnected road network. Top: hamlet for which the access clearly lies outside the main area. Middle: a very small but critical road section is administratively in another LAD. Bottom: the whole area is formed of islands

After completing this procedure, we have 326 LADs and the road network within them. To ensure the validity of the data, we tested (i) whether the network in each LAD was connected with respect to fast-food outlets and schools, and (ii) whether the network has a large disconnected component even without fast-food outlets of schools. In other word, a school or fast-food outlet that is unreachable would indicate issues with the network data. Similarly, a part of the city that is seemingly inaccessible may indicate issues in pre-processing. We found 6 LADs (less than 2% of the dataset) experiencing one of these two issues. This was mostly due to a misalignment between boundaries for governance (the LADs) and the transportation network (Fig. 5). For example, one city could be in charge of two areas, but the only road to move between them was within the boundary of another city. The six cases were manually resolved. For Tewkesbury, Windsor and Maidenhead, and Wyre, the small road fragment needed to connect the disjoint parts was re-assigned from the LAD where it fell (Cotswold and Gloucester, Bracknell Forest, and Fylde respectively). For Ashfield and North East Derbyshire, the parts that connected the ‘main’ city to a hamlet were far off, and we thus split each city into two LADs (one for the ‘main’ part and one for the hamlet). Finally, the Isles of Scilly contained roads over five disconnected islands. Since our records indicate that the islands contained no schools and no fast-food outlets, we dropped this LAD from our dataset. We thus had $326 - 1 + 2 = 327$ LADs.

### Step 3: assigning schools and fast-food outlets to road segment

The input to this step consists of the road network divided across 327 LADs, and the Points of Interest (POI) data for schools and fast-food outlets. We obtained the complete POI data for England, and filtered it using the classification scheme version 3.1 to categories 0018 (“Fast food and takeaway outlet”) and 31 (“Primary, secondary and tertiary education”) while noting that it does not include vocational schools (e.g., sailing schools, diving schools) or schools for outdoor pursuits (e.g., riding schools and equestrian centres). Consequently, ‘schools’ at this step refers to all schools but vocational or outdoor-oriented, and ‘fast-food outlets’ refers to all of them regardless of whether they include a sitting area.

**Figure 6:**
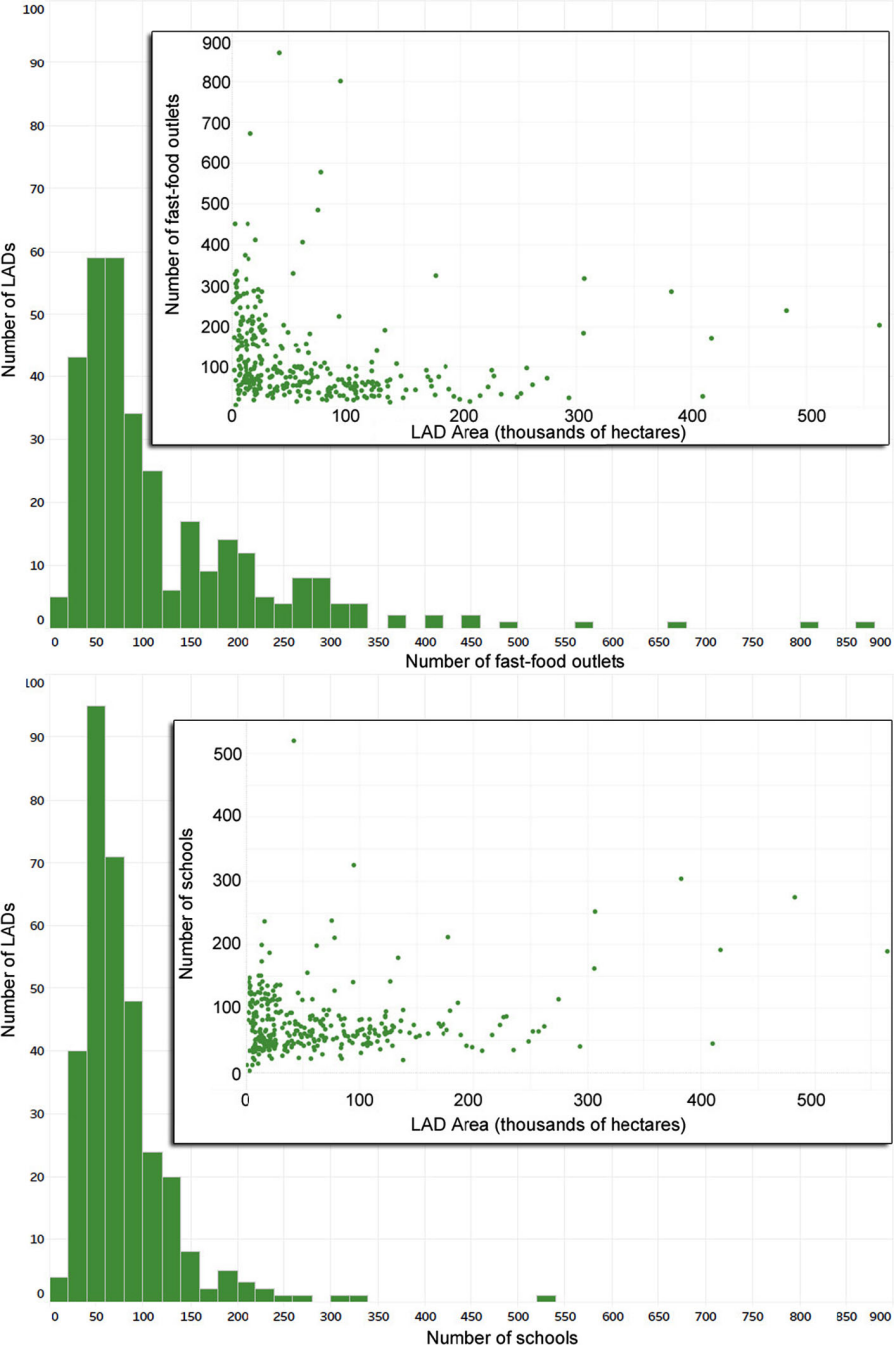
Distribution of the number of fast-food outlets and schools (*x*-axis) across LADs (*y*-axis)

The data includes easting and northing coordinates, the postal code, and a district code. Several entries had missing information, such as incomplete postal codes or no district code. We discarded such incomplete entries, representing only 0.5% of the fast-food outlets and 0.6% of the schools. For the remaining data, we assigned the entities to road segments (i.e., edges of our network) in two steps: (i) identify the LAD based on the district code, and (ii) select the edge closest to the entity. A difficulty of step (i) is that the district code provides the name of a city, and not the name of a LAD. In most cases, the LAD had the same name as the city. However, for 36 cases, there was no LAD with the city’s name. This occured for LADs that represented counties, and had several cities (e.g., County Durham includes Durham, Derwentside, Sedgefield, Teesdale, etc.). All 36 cases were resolved manually, using Google Maps as geolocation service to find the city in England, and thus identify the LAD that it fits in. After completion of step (i), we knew the LAD for 99.5% of outlets and 99.4% of schools. For each entity within a LAD, we computed its distance to all road segments of that LAD, and we assigned it to the nearest segment (i.e. with minimum distance). The resulting network (Table 5) has coordinates on the nodes, and number of fast-food outlets as well as schools on the edges. Note that we do not track the properties of individual fast-food outlets or schools, hence we only keep track of their density nearby a given road segment. The distributions of fast-food outlets and schools per LAD follows a similar pattern (Fig. 6), although we note that there are typically 0 to 150 schools per LAD whereas there is a wider possible range of fast-food outlets.

**Table 5:** Hypothetical example of data produced by step 3, showing a network where nodes have coordinates and edges count fast-food outlets as well as schools

*Nodes*		
532715 181698		
532742.771 181787.615		
532339.31689 181923.56457		
532308.7005602281 181913.6821562441		
*Edges*		
532715 181698	532742.771 181787.615	
532339.31689 181923.56457	532308.7005602281	
*Outlets/schools*		
(532715 181698, 532742.771 181787.615)	0	1
(532339.31689 181923.56457, 532308.7005602281)	2	0

### Step 4: identifying the lower layer super output area (LSOA) for each road segment

The LSOA contains statistical information. Identifying the LSOA of a road segment thus provides access to the deprivation level of this road segment. We started by excluding 5.63% of the LSOAs from the dataset because they were entirely outside of England, which is the focus of this work. Then, we identified the LSOA to which each road segment belonged. Because LSOAs were not designed to match the transportation network, we had to operate in the same way as in step 2: segments entirely within an LSOA were assigned to it, while those *partially* within the LSOA were further split. While LSOAs do not overlap, we also noted that several road segments were exactly at the boundary of two LSOAs (53,459 segments or $\approx0.8\%$ of the data), and we assigned them to both (i.e., a given edge has either one or two LSOAs).

**Figure 7:**
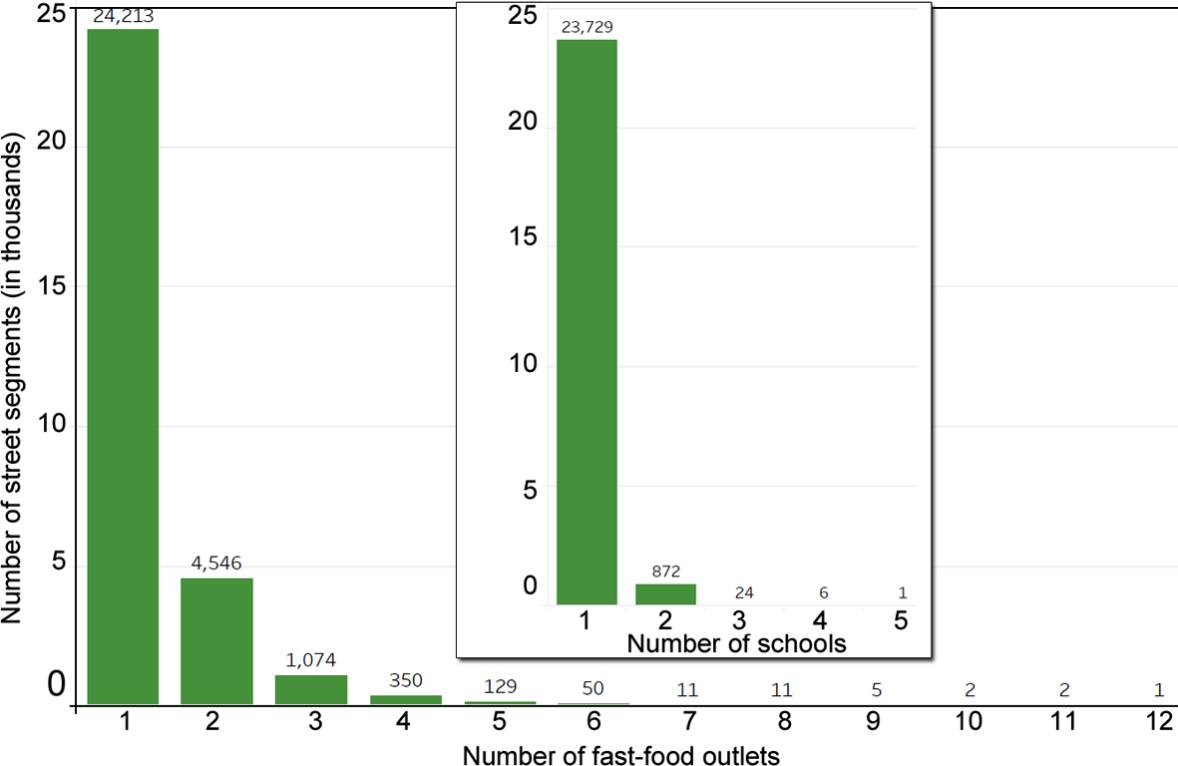
Distribution of the number of fast-food outlets and schools (inset) per street segment

This process resulted in a final network size of 6,549,676 edges and 6,102,863 nodes. This leads to an extremely low network density (${\approx} 3.51e^{-5}$), which we expect as a node is most frequently connected to two edges (since a road is stored as a series of lines) and cannot be connected to many others given the practical limitation on the number of roads that can intersect. When outlets were present on a street segment, there were on average $1.28 \pm0.69$ outlets. Similarly, when schools were present on a street segment, there were on average $1.03 \pm0.20$ schools. The distribution of schools and outlets per street segment is shown in Fig. 7.

As this is the last step that affects the existence of an edge, we also finalized spatial information about each edge at this step by computing the edge’s length (based on the Euclidean distance between its two endpoints). Computing the distance was necessary to later answer questions such as how *far* schools can be from fast-food outlets. The average edge had a length of 60 ± 70 m, with the large standard deviation due to the simultaneous presence of long non-intersecting straight roads as well as extremely small segments (e.g. for tiny portions of roads spanning two LADs, or a road with a strong curvature approximated by many small lines).

### Step 5: adding the deprivation level of each road segment via its LSOA

The Index of Multiple Deprivation (IMD), commonly refered to as ‘deprivation level’ here, is a floating-point number assigned to each LSOA. When a road segment had a single LSOA, we thus assigned it the deprivation level of its LSOA. For boundary roads assigned to two LSOAs, we could not assume that their deprivation would be more like one LSOA or the other, and thus we assigned them the average deprivation level of the two LSOAs. As in previous analyses of the fast-food outlets in England with respect to deprivation [38], we simplified the (continuous) deprivation level into tertiles. The first tertile contains deprivation levels up to 11.92 (included), the second tertile contains deprivation levels from 11.92 (excluded) to 24.845 (included), and the third tertile contains deprivation levels strictly greater than 24.845. The amount of LSOAs within each tertile could not be exactly the same (as the total was not dividable by three), hence there are 10,947 LSOAs in the first two tertiles and 10,949 LSOAs in the third tertile.

## Analytical methods

### Overview

The following two sub-sections detail why, and how we computed our results from the network assembled in the previous section. Some notation will be used throughout this section, and is introduced here. We denote a graph $G=(V,E)$ as formed of a node set *V* and an edge set *E*. The number of nodes and edges in the graph is denoted by $|E|=m$ and $|V|=n$ respectively. The ‘cost’ of an algorithm will be expressed in the worst-case, that is, as the peak resources that it needs to complete. Resources are divided into time (i.e. time complexity) and space (i.e. space complexity). The worst-case complexity is expressed using the $\mathcal{O}$ notation, showing how either the running time or space requirements grow as a function of *m* and *n*. For example, a space of $\mathcal{O}(m)$ says that we need to store ‘in the order’ of the number of edges for an algorithm (thus omitting constants). For larger networks such as ours, acceptable costs rarely exceed quadratic forms: for instance, $\mathcal{O}(n^{2})$ *may* be feasible, but $\mathcal{O}(n^{3})$ may exceed available resources. When computing distances, we chose algorithms that provide exact answers at costs less than quadratic. When computing centralities, we opted for approximation algorithms given the high cost of the exact ones. Computations were performed on the shared High Performance Cluster (HPC) Gaea at Northern Illinois University, typically using 5 nodes (each equipped with 2 Intel X5650 processors and 72 Gb RAM). Our scripts for analysis are available within the ‘Analysis’ folder at https://osf.io/gn3f2/.

### Computing shortest-path distances

The current public health context in England aims at countering the perceived proliferation of fast-food outlets around schools. The Supplementary Planning Document (SPD) can be used by local governments to enact local planning policies that affect fast-food outlets (formally defined as shop types that fall within Use Classes A5 for an SPD). While planning policies can range widely, two specific levers have received increased attention [29]. First, there can be a minimum *distance between fast-food outlets and schools*. Second, there can be a maximum clustering, by limiting the number of fast-food outlets packed in an area, which consequently would increase *distance between fast-food outlets*. In both cases, policymakers need to decide on a specific value: how close is ‘too close’ to a school? How far should outlets be from each other? In the absence of detailed data, these choices are made on a best-guess basis, reflected by a wide array of values. For instance, Islington Council set a 200 meters buffer between schools and fast-food outlets, while others used a 400 meters buffer (Warrington Borough Council, City of Bradford, Barking and Dagenham, Solihull council) [39–43]. Similarly, the clustering was set to having no more than 10% of units in an area for Gateshead Council, whereas Barking and Dagenham used a 5% limit, and Solihull imposed a 15% limit. Target areas also varied, with some using zoning to control town centers whereas others targeted specific demographics (e.g., Gateshead Council imposed restrictions in wards where more than 10% of year 6 pupils were obese) [39, 40, 44]. Consequently, a major contribution of our work is to compute the distances used in both policy levers. That is, we compute the shortest distances (i) between fast-food outlets, and (ii) between fast-food outlets and schools.

The *generic* solution to compute shortest-path distances between two objects (i.e., a fast-food outlet and another outlet or school) is typically the Bellman–Ford algorithm those time complexity is $\mathcal{O}(mn)$. In networks exhibiting desirable properties, more specific solutions can be identified. In our network, edges have a strictly positive *weight*, representing the length of the corresponding road segment. In this situation, Dijkstra’s algorithm is faster due to a time complexity of $\mathcal{O}(m+n\log n)$. While there exists an optimal $\mathcal{O}(n)$ algorithm for planar networks [45] (i.e. which can be drawn without two overlapping edges), the British road network does not satisfy this constraint due to the presence of overpasses (called flyover) including stack interchanges (when roads are above each other on multiple levels). We note that this problem does not affect all LADs: as of 2017, http://www.cbrd.co.uk/ estimated that there were less than 30 stack interchanges in the UK. Computations may thus be optimized by processing planar LADs differently than non-planar ones. However, to run distributed computations on the HPC facility, we ensured that the same version of the code was used for all inputs. Consequently, we implemented Dijkstra’s algorithm, and results were computed within approximately 42 hours.

### Relating the presence of fast-food outlets to centralities

In this section, we relate the centrality of *nodes* to the number of fast-food outlets. The motivation for this analysis is as follows. Table 1 provides a sample of ten studies, all of which investigated betweenness centrality, and most of which also used closeness centrality. Considering a street network as a transport infrastructure, a typical concern is about the flow going through the network. In the absence of real-world data on traffic flows, betweenness centrality provides a proxy to network *flows*. Specifically, it assumes that places passed by a larger number of shortest paths connecting streets are more likely to be visited. This notion has been applied to many large networks [46], and has shown good correlations with important metrics for transportation networks such as congestion [47]. Closeness serves as a proxy to *access*, by identifying how easy (i.e. distance-wise) it is to get from a street to all others. Studies have shown good correlations between closeness centrality and urban elements such as economic activities [10] (and particularly retail stores [14]) or green spaces [13]. Research on food behavior also uses access as one factor driving the choice of a food retail location for individuals [48], highlighting that individuals are more inclined to purchase food sold within up to 1 mile, although other factors such as deprivation mediate this relationship [49]. Our overall process to relate centrality and fast-food outlets is summarized in Box 1, and detailed as follows. Note that the process is applied for each LAD independently, and then results are combined across the LADs. This ensures that there is no contagion effect: the centrality of a street segment in a city depends only on the topology of this city, rather than on the city’s position in the country. In other words, by splitting the whole network into LADs and measuring centrality within each LAD, our results are not influenced by whether a city is close to the sea or border (which may lower the centrality of its streets) or situated around the middle of the country (which may inflate its centrality).


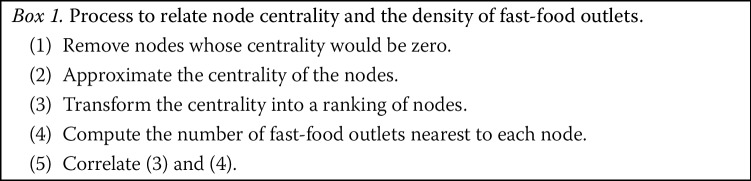


Intuitively, the betweenness centrality of a node *x* is the fraction of shortest paths between pairs of nodes in a network that pass through *x*. The closeness centrality of *x* is the inverse of the distance required, from *x*, to reach all other nodes through shortest paths. Betweenness and closeness centralities are formally stated in the two definitions below. Note that they are both centrality *indices*. For instance, for two elements *x* and *y*, if the centrality $c(x)$ is at least as much as $c(y)$, then we conclude that *x* is at least as central as *y*. As stated by Koschutzki *et al.*, “in general, the difference or ratio of two centrality values cannot be interpreted as a quantification of how much more central one element is than the other” [50]. Given that our goal is to correlate the centrality with the presence of urban elements, we do not want the correlation to be biased by wrongly using relative differences in centrality. After computing the centrality of all nodes, we thus normalize it by transforming it into a ranking.

#### Definition 1

Let $\sigma_{st}(v)$ denote the number of shortest paths between two nodes $s,t \in V$ that contain $v \in V$. Then, the *betweenness centrality* of a node $u \in V$ is given by [50]:

1 $$ c_{B}(u)=\frac{\sigma_{st}(v)}{\sigma_{st}}. $$

#### Definition 2

Let $d(u,v)$ denote the shortest-path distance between two nodes $u, v \in V$. Then, the *closeness centrality* of a node $u \in V$ is given by [50]:

2 $$ c_{C}(u)=\frac{1}{ \sum_{v \in V} d(u,v)}. $$

**Figure 8:**
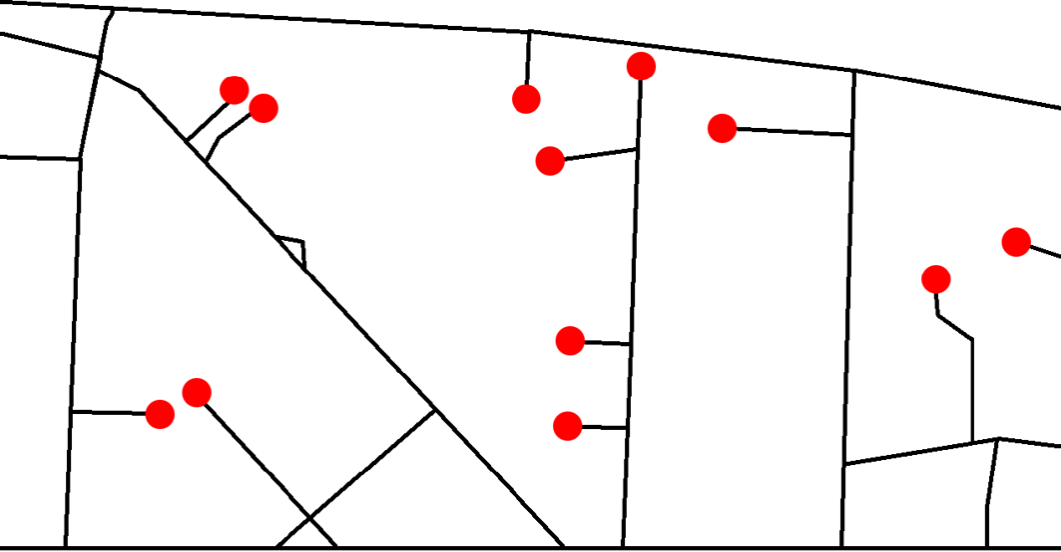
Nodes with a single edge are easy to identify, and removed before computing the betweenness centrality. This example in Adur City shows how 12 nodes (red circles) can be removed, as no path goes through them

**Figure 9:**
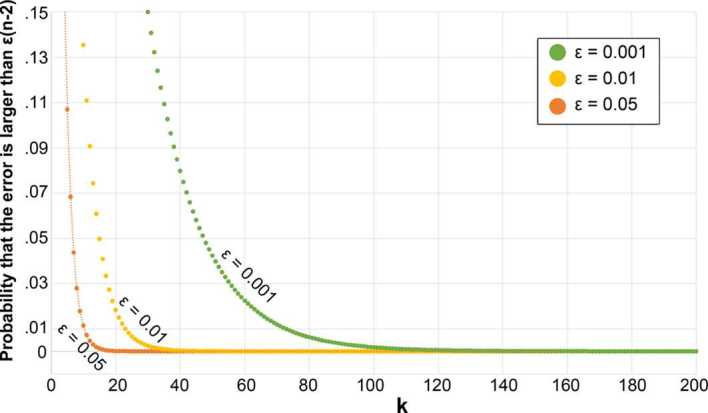
Probability that the error exceeds a target (depending on *ϵ*) for different number of pivots *k*. Computations were performed for Cornwall

**Figure 10:**
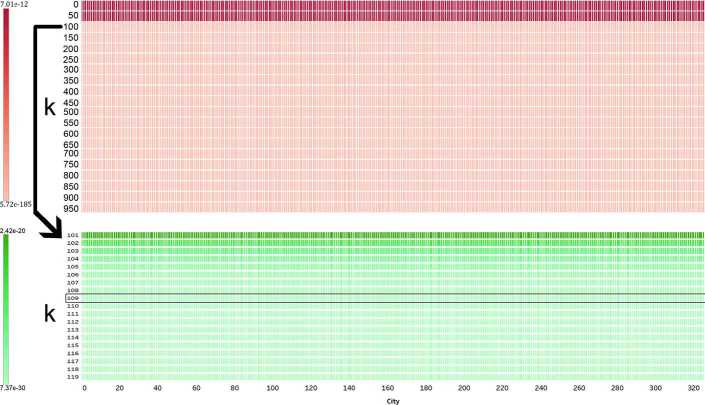
After setting *ϵ* to 5%, approximation errors depend on the number of pivots *k* (*y*-axis) and the number of nodes *n*, which varies across cities (*x*-axis). We found that the choice of city did not have a noticeable impact. Approximation error became small in the range 100–150 (top), and we chose $k=109$ (bottom; framed). Due to the wide range of values, note that scales (i.e. colormaps) are different

Computing betweenness and closeness centralities in a weighted graph takes $\mathcal{O}(n^{3})$ time with a modified Floyd–Warshall algorithm. This can be improved for a sparse graph specifically (as is the case here) by using Brandes’ algorithm which takes $\mathcal{O}(n ^{2}\log{}n+mn)$ time, but this cost remains very significant for a graph with millions of nodes and edges. We took two steps to improve it. First, similarly to Porta *et al.* [7], we excluded nodes whose centrality would be 0, *without having to compute it*. That is, for betwenness centrality, we excluded nodes with a single edge as they act as sinks and no shortest paths go through them (Fig. 8). Similarly, for closeness, we excluded unreachable nodes (since their distance to others would be infinite and their closeness tend to 0). This approach removed approximately 14% of nodes when computing betweenness, and less than 1% of nodes for closeness. We thus had to use a second step, in which we employ Eppstein and Wang’s fast approximation algorithm for betweenness and closeness [51]. The algorithm randomly selects *k* pivots, and provides the probability that estimation errors are greater than $\epsilon\times(n-2)$. A higher *k* or a lower *ϵ* would lead to more accurate results at the expense of performing more computations. We thus have to identify suitable values of the parameters *k* and *ϵ*, while noting that these choices are interdependent (Fig. 9). We set *ϵ* to a 5% error margin, and we performed a parameter sweep across all 327 LADs and values of *k* (from 1 to 1000). We identified $k=109$ as providing a good level of accuracy while keeping computational time small (Fig. 10).

After obtaining a ranking of nodes with respect to (i) betweenness and (ii) closeness, we had to correlate the ranking with the presence of fast-food outlets. Similarly to step 3 in assembling the dataset, we went through each fast-food outlet and assigned it to the nearest node (instead of the nearest edge as in step 3). Finally, we computed the correlation between the number of fast-food outlets and the ranking of the nodes, for both betwenness and closeness. Correlation values range from −1 (perfect negative correlation) to 1 (perfect positive correlation). Correlation values were computed using both the Spearman and the Pearson correlation methods, to assess whether there was a monotonic (for Spearman) or linear (for Pearson) relationship between the presence of fast-food outlets and the centrality metric.

## Results

**Figure 11:**
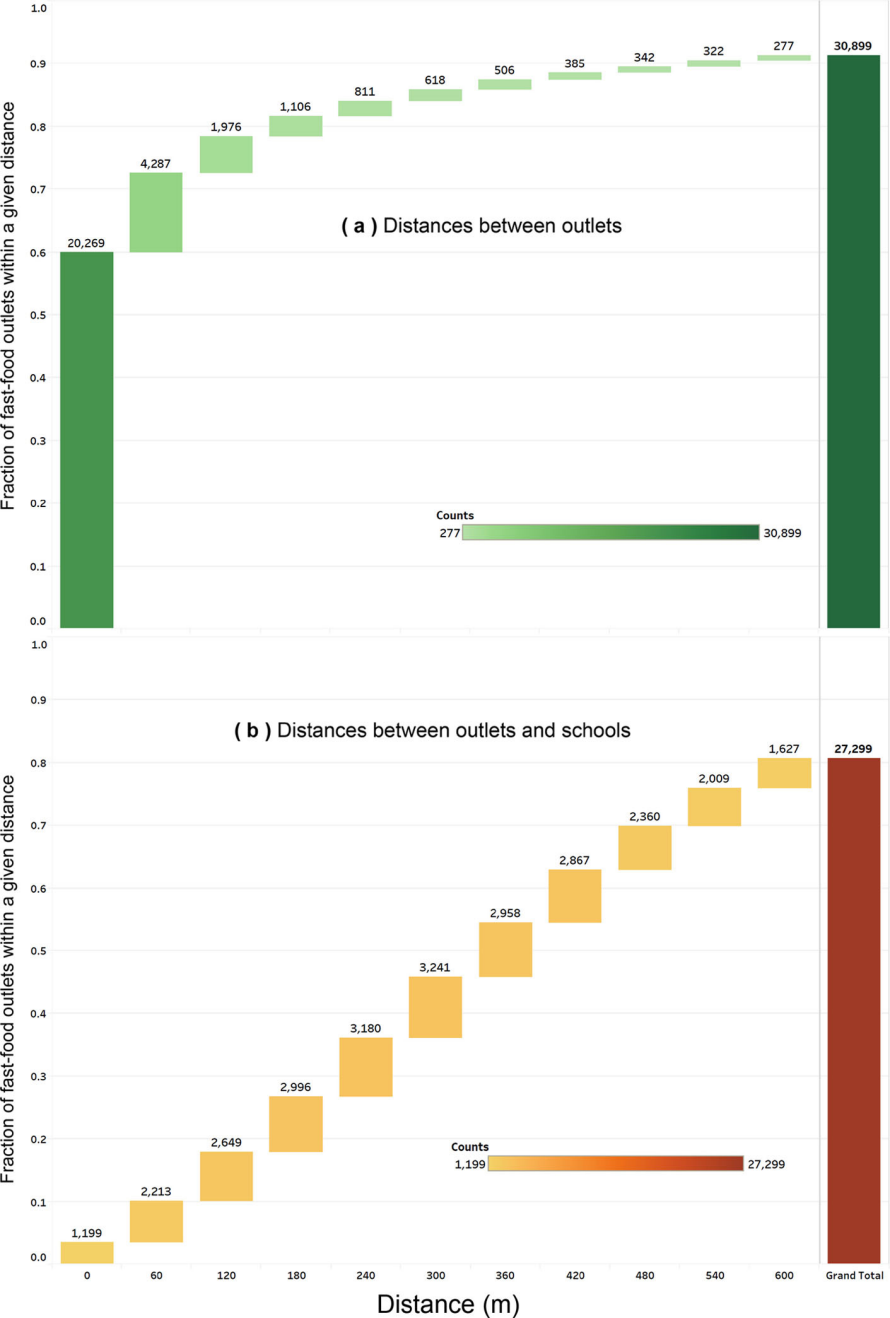
Distribution of distances (in meters) between outlets (**a**) as well as between outlets and schools (**b**). The *a*-axis goes up to 600 m as it is the largest value encountered in current zoning policies regarding fast-food outlets and schools in England. *An expanded version of the figure also using Euclidean distances is available on* *https://osf.io/gn3f2/*

The datasets produced by our analysis (previous section) are available within the ‘Results’ folder at https://osf.io/gn3f2/. We computed the distributions of distances between fast-food outlets and (i) the nearest fast-food outlet, as well as (ii) the nearest school. Based on these distributions (Fig. 11), we can make the following observations:

- Fast-food outlets are very strongly clustered. Most of them are located either on the same spot or within a few dozen meters (60% of the data falls within 0 to 60 meters). Using a 120 m buffer suffices to capture almost 80% of the outlets.
- While outlets are strongly clustered around each other, they are much less clustered around schools. Less than 5% of outlets are found within 60 meters of a school (compared to 60% with respect to other outlets), and less than 20% of outlets are found within 120 m of a school (compared to 80% with respect to other outlets).
- The widest buffer of 600 m around a school would capture over 80% of existing outlets, while the other classic buffer of 420 m would capture about 65%. This shows that doubling the buffer does not double the number of outlets included.

In Fig. 11, the number on top of the leftmost bar is the number of outlets that are about 0 m away from the nearest location (outlet/school). The numbers above the next bars would be the number of outlets that are more than the upper bound of the previous bar but less than or equal to the number below the bar. For instance, the number in the distance from outlets to schools (Fig. 11(b)) counts up the number of outlets within a certain distance to the closest school. Both parts of Fig. 11 track the number of outlets, the difference being whether the distance is from the closest outlet (Fig. 11(a)) or the closest school (Fig. 11(b)). To examine whether the distance computed by the street network was different from the distance ‘as the crow flies’, we also computed the distance using the Euclidean distance (provided as additional figure on https://osf.io/gn3f2/).

**Figure 12:**
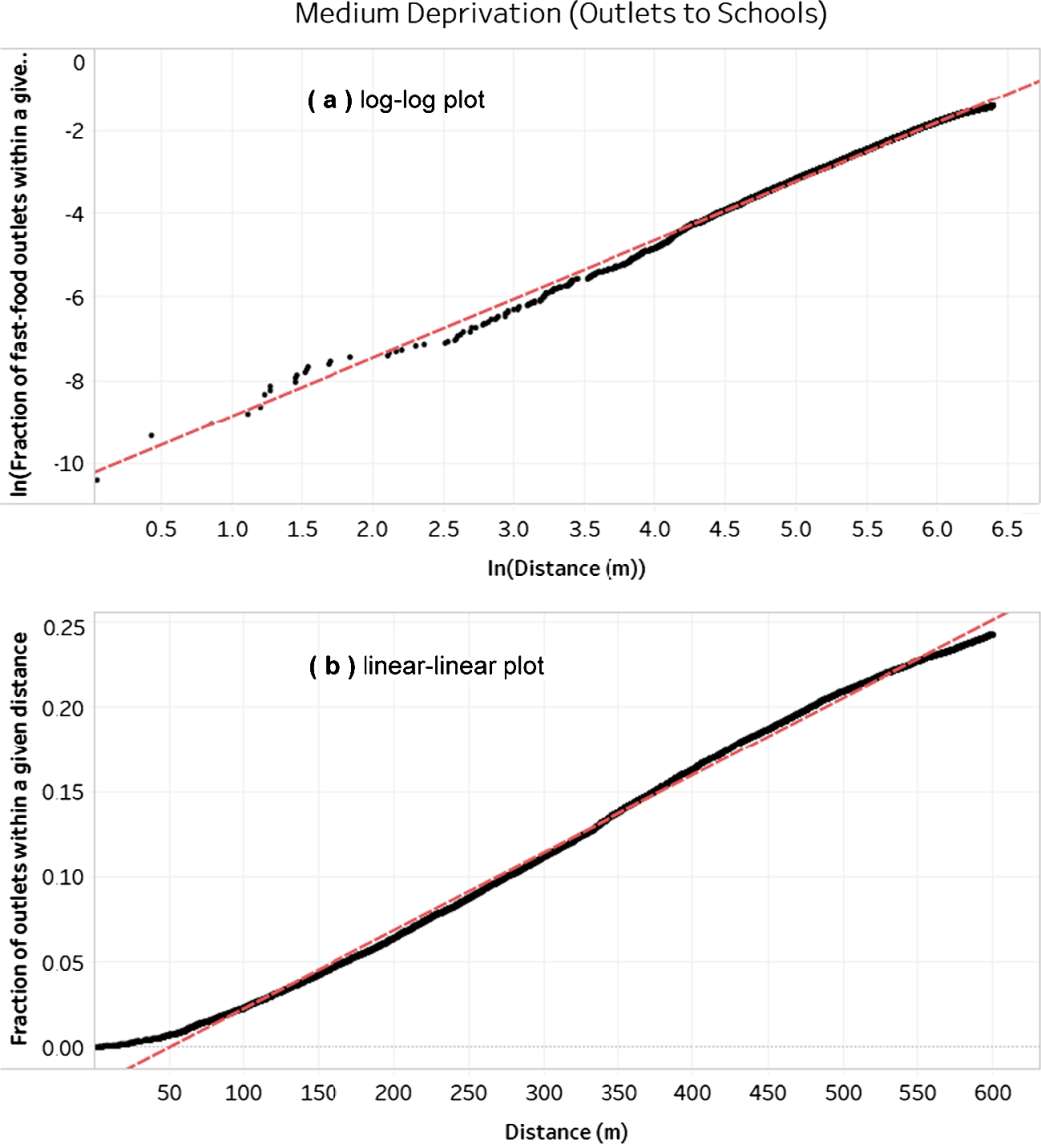
Fit between the analysis output (transformed from discrete to continuous) using a log-log plot (**a**) and a linear-linear plot (**b**), for a medium level of deprivation, for distances between fast-food outlets and schools. *An expanded version of the figure showing all four combinations of linear and logarithmic scales is available on* *https://osf.io/gn3f2/*

We further investigated the relationship between distances from schools and the fraction of fast-food outlets found, in general as well as across levels of deprivation (e.g., Fig. 12 shows two of the four different fits computed for a medium level of deprivation). After transforming our discrete distribution into a continuous one, we fitted four different types of curves (Table 6). We obtained an almost perfect fit ($R^{2} = 0.99$) with either a linear relationship or a power-law (i.e. a line on a log-log scale). It is of particular interest to observe that the exponent of the power-law (i.e. the slope of the line on the log-log scale) was 1.4 across all four categories of deprivation shown in Table 6. The values of the exponent only start to differ at two decimal places depending on the level of deprivation. There are thus two competing hypotheses on the relationship between distances from schools and the fraction of fast-food outlets: either linear or power-law. The power-law hypothesis may be supported by two arguments: the existence of a power-law in many networks shaped by human activities [52], and the presence of scaling laws (which here may govern the distribution of the number of outlets with the distance) often justified by the interplay between the fractal properties of the cities and the behavior of inhabitants [52–54].

**Table 6:** Fits across levels of deprivation and scaling of the axes

*Deprivation*	*Distance scale (x-axis)*	*Fraction of outlets scale (y-axis)*	*p-value*	$R^{2}$ *of a linear fit*
*Overall*	linear	linear	<0.0001	0.996989
		log		0.776541
	log	linear		0.810363
		log		0.993964
*Tertile 1*	linear	linear		0.99789
		log		0.786763
	log	linear		0.828365
		log		0.991716
*Tertile 2*	linear	linear		0.996447
		log		0.782838
	log	linear		0.795886
		log		0.994874
*Tertile 3*	linear	linear		0.996617
		log		0.769842
	log	linear		0.813284
		log		0.992019

**Figure 13:**
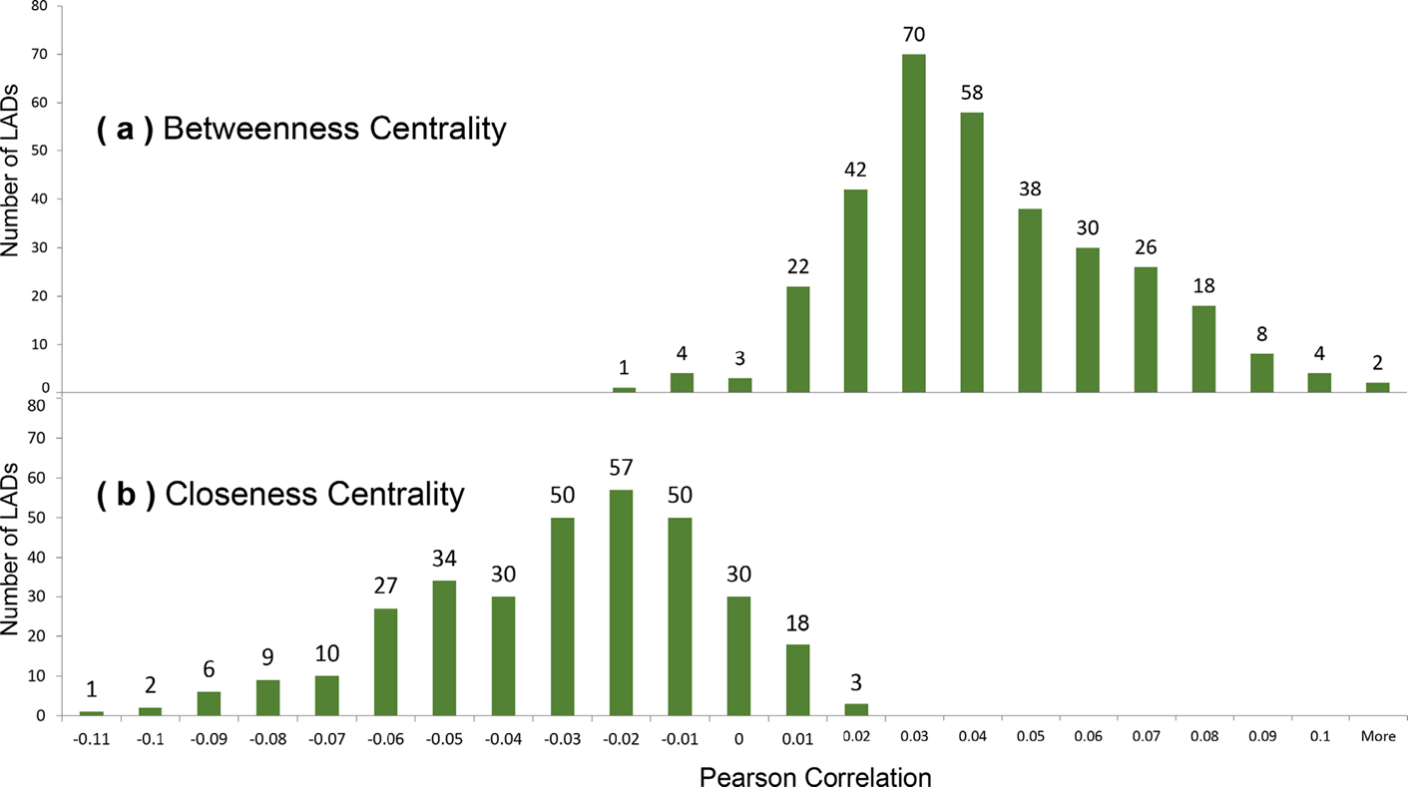
Distribution of Pearson correlations between the density of fast-food outlets and (**a**) betweenness centrality or (**b**) closeness centrality

**Figure 14:**
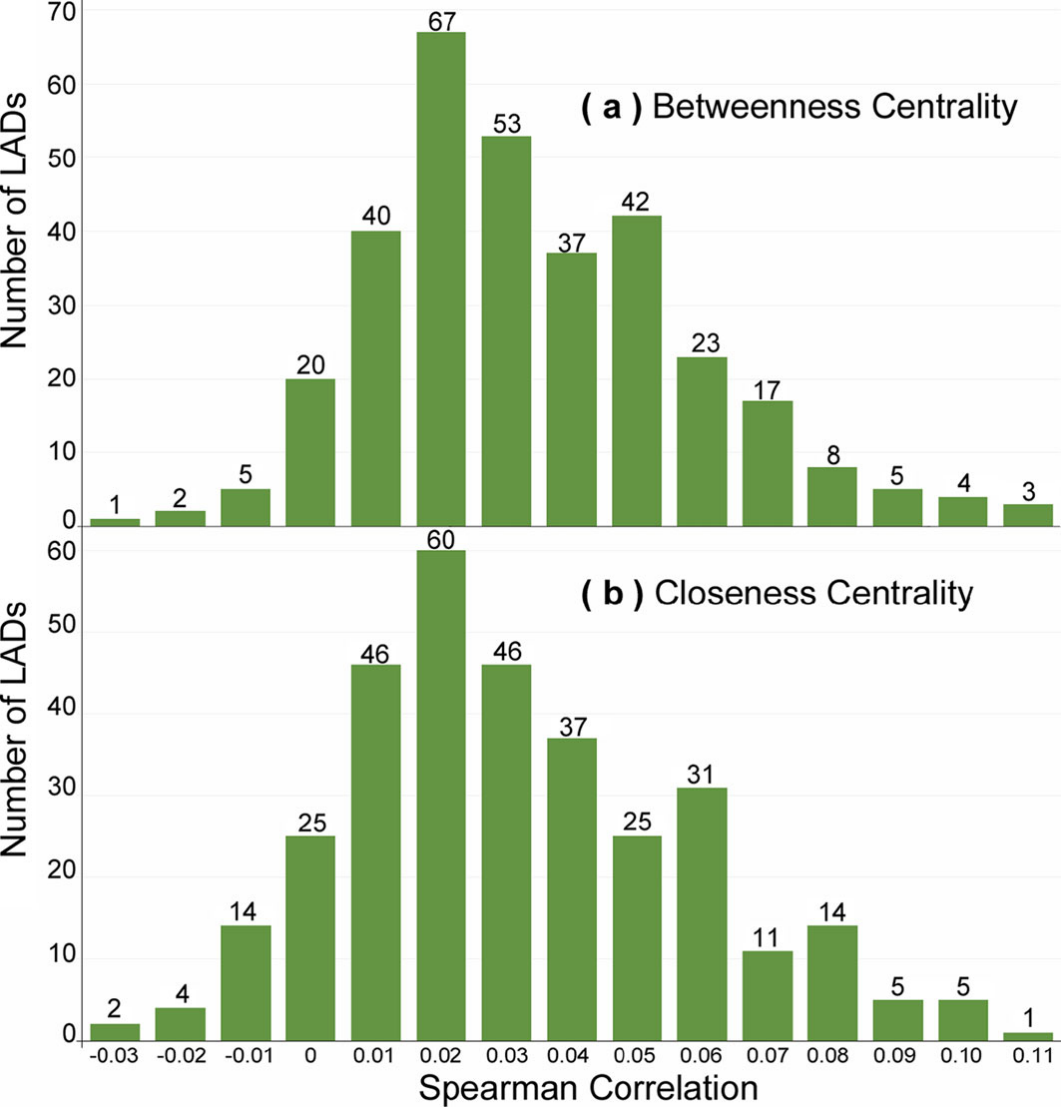
Distribution of Spearman between the density of fast-food outlets and (**a**) betweenness centrality or (**b**) closeness centrality

The correlation between centrality and the presence of fast-food outlets is shown in Fig. 13 for the Pearson correlation, and Fig. 14 for the Spearman correlation. We observe that almost all of the data falls within the range $[-0.1, 0.1]$ in which we conclude to the absence of a correlation. While three points fall outside this range, they are still at a very low level of correlation and may be outliers.

## Discussion

While network analyses of retail activities have been performed at local scales (Table 1), our study is the first to do it over an entire nation. This was made possible by obtaining and linking very detailed datasets, including the position of all outlets as well as the complete road network. Our focus is on fast-food outlets, and their relationship with schools. Research has suggested that this relationship is mediated by the level of deprivation [31–33], which we have included in our dataset to examine our findings across levels of deprivation.

Our first research question was to identify the distances between fast-food outlets and (i) other outlets as well as (ii) schools. This was motivated by the pressing need for a national evidence base to either (i) increase distances between fast-food outlets by limiting clustering, or (ii) create a buffer around schools. The 2011 guidance from the National Institute for Health and Clinical Excellence recommended that local authorities regulate the number of fast-foods in specific areas, such as within walking distance of school [25]. The 2013 Academy of Medical Royal Colleges’ report advocated to “reduce the proximity of fast food outlets to schools, colleges, leisure centres and other places where children gather” [55]. However, neither could say exactly by which distance to reduce it, and what number of outlets would be affected, as this analysis was not previously available. Following these recommendations, several local authorities have started to use planning as a tool to address childhood obesity. As summarized by Peter Wright, an emerging view is that improving nutritional quality

“is not an issue that will be satisfactorily resolved by voluntary improvement, education, advice or any other “easy” intervention. Without political will and a determination to limit the proliferation of takeaway food businesses we are unlikely to make any meaningful impact on the impact of poor diet on significant parts of the population.” *Peter Wright, Gateshead Council, Centre for Diet and Activity Research (CEDAR), ‘Neighbourhood food environments, diet and health: research policy meeting’, Nov. 4th 2014, Cambridge, UK.*

Given the reality of having to address childhood obesity, local authorities thus had to make assumptions about what distances were the right ones and what effect would be obtained. This illustrates the two unknowns: what distance should we use, and how many fast-food outlets would it capture? The Takeaways Toolkit, considered to be one of the reference documents to assist with designing regulations, has previously emphasized the need for more evidence since such planning measures “have not yet been evaluated, and the impact on obesity and other health issues remains unknown”. This study contributes to the creation of robust evidence through our national-scale analysis of distances. We found strong spatial clusters of fast-food outlets (Fig. 11(a)): most fast-food outlets were within a few dozen meters from each other, and 80% of them were within 120 meters. However, clusters around schools were significantly weaker (Fig. 11(b)): less than 5% of outlets were within a few dozen meters from schools, and going as far as 120 meters captures less than 20% of them (compared to 80% when using other outlets as referential). This finding is in contrast to previous studies finding strong clusters around schools. This difference may be explained partially by context, as previous analyses were conducted in Scotland, New Zealand, or the United States instead of the United Kingdom [56–58]. Our data can also inform authorities having implemented buffers around schools about the average fraction of outlets that may be captured: the 200 meters buffer for Islington Council [43] may impact a third of the outlets (based on national averages), while the 400 meters used by others may impact half of the outlets [39–41, 43]. This suggests that increasing the distances between fast-food outlets may create more disruptive changes in the foodscape. However, like many upstream interventions, being disruptive can be both an opportunity (to avoid concentrated obesogenic environments) and a challenge (as many actors are concerned and a high political capital may be needed to enact such changes). Our last contribution regarding fast-food outlets and schools is to examine their relation across levels of deprivation. We found that a scaling law was most likely to govern the relationship between distances from schools and the fraction of fast-food outlets. The underlying explanation for this scaling law would need to be explored in a follow-up study, for instance by using the concept of *fractal dimension* of a city. As shown by the comprehensive study of Ribeiro *et al.* [52], the concept of fractal dimension is related to urban metrics of infrastructure (e.g., using the number of fast-food outlets as infrastructure variable) and the decaying influence of one node over another.

Our second research question was to investigate the relationship between network centrality and the density of fast-food outlets, thus taking previous local studies (Table 1) to a national scale. While previous studies found strong correlations between centrality and economic activities ($R^{2} = 0.61$ [10], or $R^{2} = 0.651$ [14]), we found no correlation using either Spearman or Pearson methods: the correlation was close to 0 for 324 out of 327 areas, and only marginally beyond −0.1 or 0.1 for 3 areas (Figs. 13 and 14). This suggests that, either at the national scale or at the scale of our areas, closeness or betweenness centrality were not a sufficiently strong factor to explain the location of outlets.

There are several possible explanations for the absence of correlation between centrality and number of fast food outlets. The assumption for correlation is that a central street segment would be more ‘advantageous’ for fast-food outlets. However, there are spatial differences in underlying demand density for such outlets. It may thus be difficult to reduce the location choice of an outlet to a matter of street topology, given that locations are confounded by heterogeneous spatial demands in the population for services [59]. Several factors have long been provided in the literature to explain the location of businesses, either from the businesses’ viewpoints or from a customer perspective. For instance, spatial differentiation from the competition helps to avoid price rivalry and increase chances for monopoly rents. Consequently, not all stores may want to occupy a position with a high flow (betweenness centrality) or that easily reaches other destinations (closeness centrality). In addition, separation increases market coverage, which has historically been shown to play a role when travel costs are important to customers [60] or if demand changes over time [61]. Centrality may thus have to be conceptualized as a competitive process, which is captured by a few centrality indices such as the centroid [50]. However, studies on street networks and the presence of retail activities predominantly use centrality indices from the Multiple Centrality Assessment (MCA) method created by Porta and colleagues [7], which includes betweenness and closeness centrality but does not encompass the centroid method or competitive centralities. While the application of the centroid method would be an interesting alternative, it still may not fully capture the presence of fast-food outlets as their location is driven by a balance of competition and attraction (e.g., to influence customers’ ability to remember locations and contrast offers [59]).

While our study combines large datasets from the national mapping agency with other governmental sources, there are nonetheless limitations to this work. The first and main limitation is that our work is primarily of benefit to quantify economic impacts, whereas the translation into health outcomes would require a simulation. That is, our work provides estimates about how much of the food landscape may be impacted given current distances. This is the exposure to foods. Our study does not directly explain what health consequences may be obtained by a policy. This requires understanding how changing the exposure to foods would impact their utilization by children, and linking the change in diet to a change in obesity. An agent-based model could build on our work to simulate how agents (i.e. children) utilize the food environment [62], which would require detailed datasets on the food environment and childhood obesity such as the Child Obesity and Excess Weight dataset.^6^

Second, the location of outlets and schools is highly accurate but may not be perfect, as previous analyses have found the accuracy of the location database to range from 81% to 100% [30]. This creates a small margin of uncertainty on our results, but would not affect our broad conclusions on the lack of correlation between fast-food outlets and betweenness/closeness centrality or the much stronger clustering between outlets compared to outlets and schools. Third, while we used the most common forms of centrality from previous studies, there are many other forms. In particular, authors have also proposed using straightness [7, 9–11, 13, 14], or less common notions such as the cumulative number of turns or intersection crossings to reach destinations [8]. These metrics could also be approximated from our dataset, since each intersection or turn led to divide a road into another edge. However, the scale of our dataset raises the problem of efficient algorithms, and not all centrality metrics are supported by approximation algorithms (whose approximation factor is well-known or controllable). In addition, as there are dozens of centrality metrics [50], implementing and trying many would be a significant endeavour while not being necessarily the most informative. Indeed, it may be that several metrics taken *independently* exhibit low or no correlation, but *together* they may be more informative. In our future work, we plan to explore the combination of metrics that best explain the location of fast-food outlets. In addition, while this work provides national evidence regarding the strength of the *association* between schools and fast-food outlets, it cannot be used to make inferences about *causation*. Our next study will focus on causation, examining how different factors may successfully replicate the location of fast-food outlets.

## Footnotes

1 https://www.ordnancesurvey.co.uk/opendatadownload/.

2 https://www.ordnancesurvey.co.uk/opendatadownload/.

3 https://www.ordnancesurvey.co.uk/business-and-government/products/points-of-interest.html.

4 https://data.gov.uk/dataset/lower_layer_super_output_area_lsoa_boundaries.

5 https://www.gov.uk/government/uploads/system/uploads/attachment_data/file/467774/File_7_ID_2015_All_ranks_deciles_and_scores_for_the_Indices_of_Deprivation__and_population_denominators.csv/preview.

6 https://data.gov.uk/dataset/child-obesity-and-excess-weight.

## Abbreviations

IMD: Index of Multiple Deprivation, which takes into account multiple variables such as takes employment and education
LAD: Local Authority District, a division of the UK based on local governance
LSOA: Lower layer Super Output Areas, a division of the UK based on census data
HPC: High Performance Cluster, the equipment used to perform our computationally intensive analysis
POI: Points of Interest, database for this study
SPD: Supplementary Planning Document, used by local governments to enact planning policies
UK: United Kingdom, focal country for this study
